# Mobile education in autopsy conferences of pathology: presentation of complex cases

**DOI:** 10.1186/1746-1596-1-42

**Published:** 2006-11-09

**Authors:** Kerstin Schrader, Trong-Nghia Nguyen-Dobinsky, Klaus Kayser, Thomas Schrader

**Affiliations:** 1 PhysioAkadem – Schule für Physiotherapie, Berlin, Germany; 2 Multimediazentrum MMC Charité Universitätsmedizin Berlin, Germany; 3 Institut für Pathologie, Charité Universitätsmedizin Berlin, Germany

## Abstract

**Background:**

*MeduMobile *was a project to develop and evaluate learning scenarios for medical students and teachers by use of video communication and notebooks. Its core part was assigned to various medical routines, conferences or meetings such as doctor-patient bedside conversation. These were filmed by video teams and broadcasted live via the WLAN of the Charité campus to course participating students. One type of the learning arrangements was the autopsy conference as an on-call scenario.

**Materials and methods:**

The *MeduMobile *project consisted of two main compartments: the regular seminar event which took place every week or month, and the on-call event. For an on-call event the students were informed two hours before the lesson's start. A mobile video team organised the video conference via a specific *MeduMobile *seminar system. This software offered the students to log. The *MeduMobile *seminar system is based on the Windows operating system and realises an extended video communication via WLAN. Thirteen access points were implemented at the Charité Campus Virchow Klinikum and Campus Mitte. A questionnaire was developed to investigate in the response and learning effect of the mobile seminar system.

**Results:**

During the *MeduMobile *project 42 video conferences with (cumulative) 145 participating students took place. Four autopsy conferences could be organised as on-call scenarios within this project. A prospective, not randomised follow-up study was included 25 students of the 1^st ^– 6^th ^clinical semester. According to the answers, professional reasoning, professional performance, sustainability, and the complexity were broadly accepted by the students.

**Discussion:**

In principle, the MeduMobile realised an interdisciplinary case presentation using video conference and web page. The evaluation indicates a high acception of such complex case presentation with multidisciplinary settings. The use of the notebooks in mobile learning enables an interconnective training and promotes a complex learning.

## Background

A new law for medical approbation (board examination) was enacted in Germany on April 27, 2002. New aims and main focuses for medical training and education were established by the government [1]. These courses were supposed to:

 be more relevant for medical practise,

 ascertain a close relationship between theory and practise and

 improve the interdisciplinary teamwork.

This law determines for the first time the content of medical education, and, in addition, the methods of knowledge transfer. The government demands from the students to acquire skills for life time education that include soft and social skills such as personal responsibility, independence and learning aptitude.

Due to the new law the demands on universities were increased; however, the financial and personal frame were not. Especially the economic conditions were not adjusted to the new duties. For example, today in door patients have a significant shorter bed-time than some years ago. As a consequence, bed-side teaching is more difficult with patients who stay on a ward no longer than two or three days.

For medical education, therefore, new and more effective teaching methods have to be established. Various forms of teaching scenarios were offered at the Charité, University Hospital Berlin, in the last time, e.g. eLearning via the Internet or mobile learning.

The project named *MeduMobile *took place between the 1^st ^April 2003 and 31^st ^March 2004 within the framework of the Notebook-University project of the Federal Ministry of Education and Research, Berlin, Germany. The purpose of the *MeduMobile *project was the development and evaluation of learning scenarios for medical students and teachers by use of video communication and notebooks [4]. Its core part was the use of notebooks with emphasis on multimedia, interactive close-to-patient and work reality learning. Different medical disciplines participated in this project and offered various scenarios. These included dermatology, cardiology and pathology. Medical routine examinations, conferences or meetings, for example, doctor-patient bedside conversation or autopsy conferences were filmed by video teams and broadcasted live over the WLAN of the Charité campus to the course attending students. This article describes the interactive and multidisciplinary *MeduMobile *project and reports the benefit as shown by evaluation data collected form surveying our students.

## Materials and methods

The *MeduMobile *project consisted of two main compartments: the regular seminar event, which took place every week or month, and the on-call event. For an on-call event the students were informed via SMS, email or call two hours before the beginning of the lesson. Clinical meetings such as the autopsy conferences at the Institute of Pathology, Campus Charite, Univerity Berlin were prepared and offered as an on-call event.

In order to prepare the autopsy meeting for the video conference, a didactic concept with a schedule and organisation of communication with the students was developed. A group of tasks were prepared to be given to the students. A moderator presented the event and explained the details of the autopsy.

**Figure 1:**
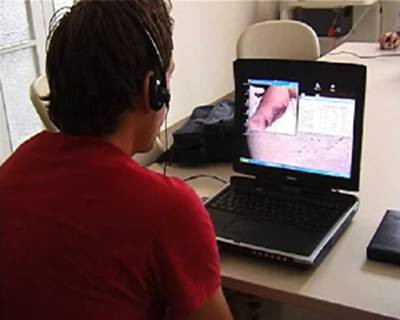
Student in a MeduMobile session.

A mobile video team organised the video conference. It used a self developed video conference system: the *MeduMobile *seminar system. This software offered various functions for online seminars: after the students had logged on the instructor received a message on his monitor. The students followed the session (figure 1), the instructor and moderator guided and communicated with them. They discussed the results together, explained the clinical data and medical history and correlated them to the morphologic findings.

**Figure 2:**
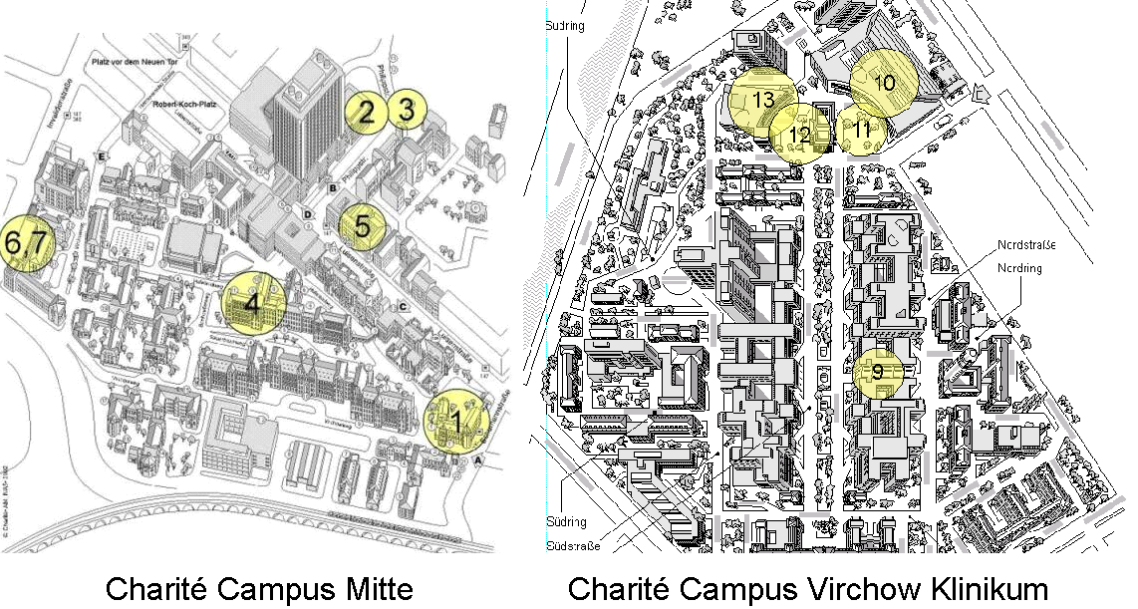
WLAN Access Point of Charité Campi.

**Figure 3:**
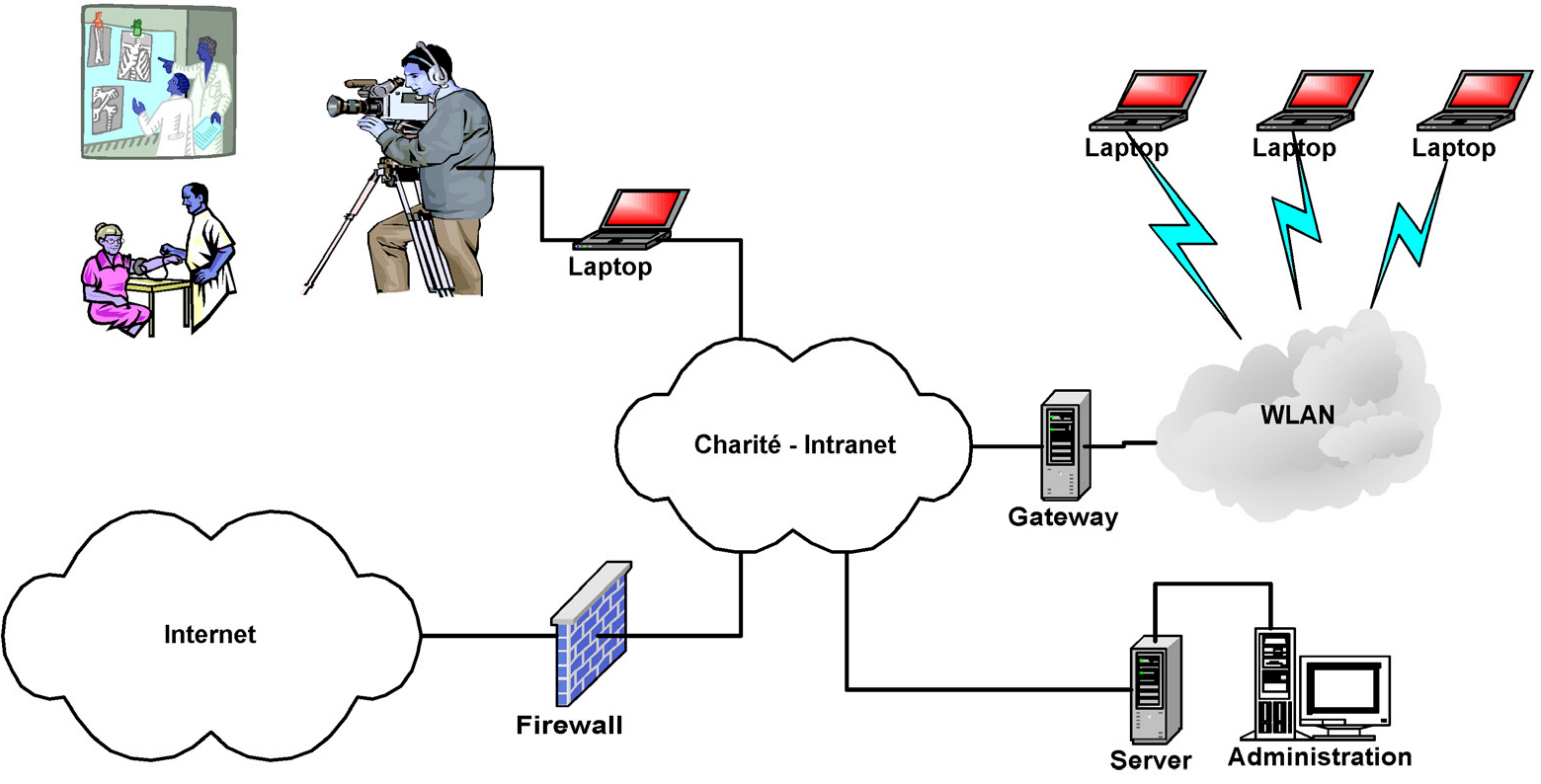
The MeduMobile-Network integrated in the Intranet of the Charité.

The *MeduMobile s*eminar system is based on the Windows operating system and realised the extended video communication via WLAN. Thirteen access points at the Charité Campus Virchow Klinikum and Campus Mitte were implemented (figure 2). Due to the protection of data privacy the WLAN operates in a separate network connected to the regular intranet of the Charité by a gateway server in order to prohibit a direct data access. Only the official web pages of the clinical partners and the internet outside the Charité were opened for the students (figure 3).

The main aim of this study was the didactical added value of case study and presentation of complex, interdisciplinary cases. Therefore a prospective, not randomised follow-up study was carried out with 25 participating students (20 male, 5 female, between the 1^st ^– 6^th ^clinical semester). The *MeduMobile *events were evaluated by use of a self-developed questionnaire. To minimize a bias towards mid scale responding a list of statements was given to the students to agree on a 4-point scale. The restricted scale options allowed a distinct differentiation of answers and therefore a clear statistical analysis, even in a small sample.

## Results

The described notebook technology in combination with a video conference system should realize and analyze complex case studies. Our questionnaire was designed to study the impact of these studies on the learning experience. The following distinctions regarding the case study were made in order to obtain more impact details:

• Professional reasoning,

• Professional performance,

• Sustainability and

• Complexity.

**Figure 4:**
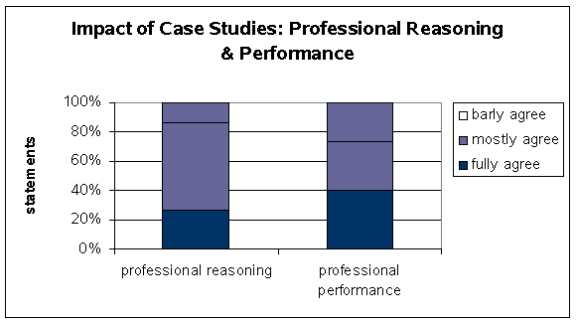
Impact of Case Studies: Professional Reasoning & Performance.

**Figure 5:**
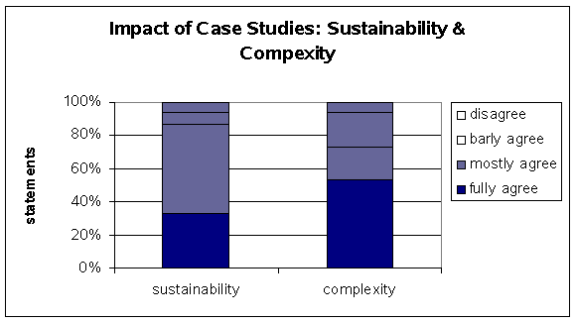
Impact of Case Studies: Sustainability & Complexity.

**Figure 6:**
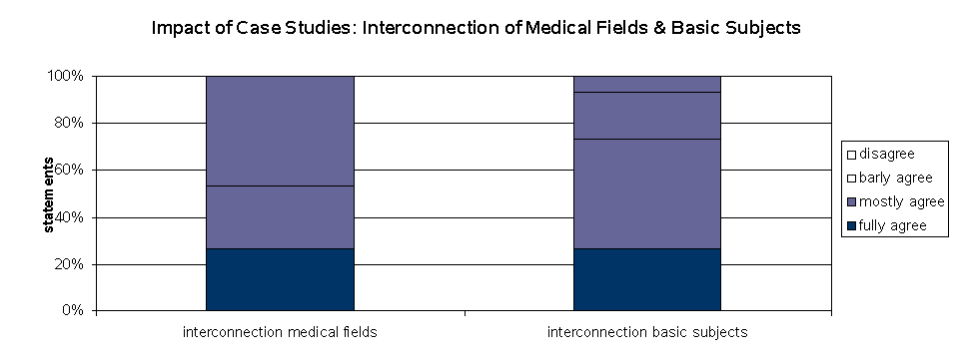
Impact of Case Studies: Interconnection of Medical fields and Basic Subjects.

The data are depicted in the figures 4, 5, 6. The positive response patterns are grouped between 80% – 90%.

### Professional Reasoning

To measure the influence of the complex case presentation on the professional comprehension was a main advantage of the project. This influence was measured by the questionnaire. More than 85% of the students declared that the interdisciplinary and electronic case presentation including the patient's history and acquired images is more appropriate than conventional seminars in terms of learning and education (figure 4).

### Professional Performance

The students had the opportunity to observe the professional action and interdisciplinary communication during the autopsy performance and conferences. The answers in the questionnaire clearly demonstrate the significance of these sessions for the students. Nearly three quarters of the involved students noted an excellent professional performance. As a result the students received high level training for the daily clinical work (figure 4).

### Sustainabilty

In our study we also investigated the sustainability of the case presentation. The students were asked about their impression of the *MeduMobile *session. About 87% of the students declared that the online session was more valuable and impressive than conventional lessons (figure 5).

### Complexity

A complex case is defined by possessing more than one diagnosis and two ore more classical medical disciplines. The participating students were also asked to state their opinion about the importance of complex case presentations in the questionnaire. The following example might illustrate the situation: An autopsy of a 65-year-old patient was performed revealing the main diagnosis of non small cell lung cancer as well as the additional diagnoses of cardiac insufficiency and compensated renal insufficiency. The patient was treated by surgery and died in the Department of Internal Medicine.

About 70% of the students accepted and appreciated this kind of case presentation including the performance of the case's data base and web pages' information of the involved institutions (figure 5).

The questionnaire also investigated the transparency between the involved medical specialties of the complex case studiest. More than 50% of the students understood the interconnection better in the *MeduMobile *case studies in comparison with the standard seminars they had attended so far. Asked whether the complex case studies demonstrated an improved association between basic theoretical medical knowledge such as anatomy or physiology and the applied medical treatment almost 3 out 4 students agreed to this statement (figure 6)

## Discussion

The *MeduMobile *project is embedded in the endeavours of the government to improve and modernize the education provided by medical universities, high schools and universities of applied sciences. The main aim of these efforts is the encouragement of livetime learning and essential professional skill [5,6]. The fast development of knowledge in medicine is a great challenge, and the methods of knowledge transfer have to be adjusted to these changes [7]. The new law of medical approbation is an expression of the efforts to increase the efficiency of education and to respond to the necessities of an information society.

The use of modern technologies and multimedia influences and creates new teaching and educational scenarios in the form of online and offline sessions. The derived applications of multimedia performance at the Charité Campus, University of Berlin include the *MeduMobile *project and other examples of eLearning environments such as *Meducase *[2] or *Sympol *[3].

Mulimedia performance in continuous education posseses several advantages. These include the possibility of :

 cross-linking of content, topics and knowledge,

 individualisation of the teaching/learning process and

 continuous education in an asynchronous manner [8,9].

During the *MeduMobile *project 42 video conferences with (cumulative) 145 participating students took place [4]. Four autopsy conferences could be organised as on-call scenarios within this project. The students were asked to participate and automatically informed through telephone call, email or a SMS by the *MeduMobile*-Server.

**Figure 7:**
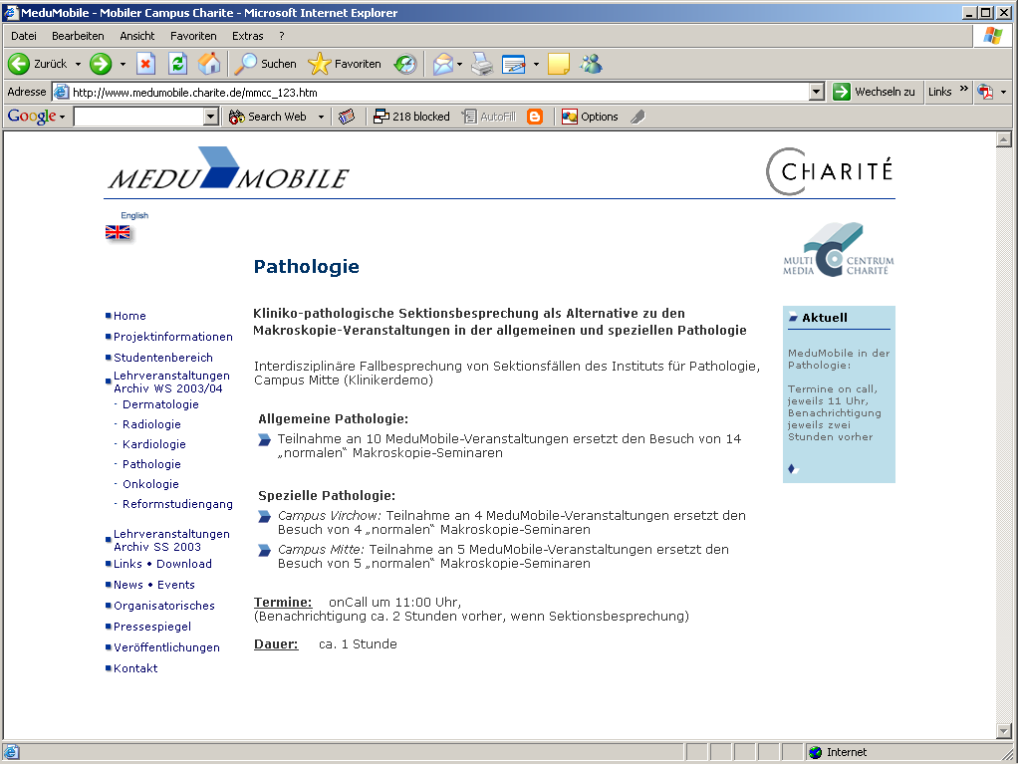
Website of the MeduMobile Project with presentation of information about the Pathology sessions.

It was essential for the *MeduMobile *project to realise an interdisciplinary case presentation using video conference and a web page as an additional information resource (figure 7). The evaluation displays the high impact of such a presentation of complex cases with multidisciplinary settings. Professional reasoning and performance as well as sustainability were especially supported by this type of educational arrangement in concordance with the literature [6,10,11].

In addition to the interdisciplinary aspect, the possibility of interaction between the student, instructor and moderator is also of significance [12]. This feature of modern multimedia eLearning environments is the most crucial one due to the necessity of achieving an appropriate level of interaction [13,14]. In the *MeduMobile *project a direct communication based on the video conference system was achived. However, other potential advantages could not be fulfilled. These include:

The session of autopsy conferences had strict schedules and supported only a synchronous teaching process.

The roles and course in an autopsy conference are fixed and cannot be changed by the participating students. Therefore, the aspect of a self-determined learning being basically a property of multimedia in eLearning [9] cannot be directly implemented by use of a video conference system.

In summary, the video conferences can be successfully applied in autopsies via WLAN because of the following reasons:

1. An autopsy conference has no direct medical implications for the participants. Normally the students observe the clinical – pathology conference directly in the autopsy lecturehall. In contrast to understanding the clinical history and the morphological findings the practical performance of an autopsy is not the explicit learning target. Thus, the students can receive this information by video conferences.

2. An autopsy conference is an interdisciplinary event. Both – the involved physicians and the pathologist – discuss the clinical course, the morphological changes and the causes of death. The students can observe both sides of the daily routine: they receive information and data from the clinical point of view and they can see the "hard" data in terms of organ changes.

3. Technically it is possible to record the organ abnormalities in high quality including the explanations of the pathologist to the clinicians. An instructor and moderator team can support the learning process by questions and tasks. They can explain complex changes in more detail and can review the whole case for the students afterwards.

4. The operating expenses for preparation and realisation the described education modules are reasonable. They mainly include the costs for a flexible *MeduMobile *team responsible for the technical management and the participating instructor and moderator.

5. Relations between anatomy, physiology, and pathophysiology can be supported by virtual graphs and video sequences.

6. The use of notebooks in a mobile educational environment enables an interconnected training and promotes complex learning. Mobile Learning is a promoting additative to the eLearning-environment including the Virtual Microscope in pathology [15,16].
